# The Effects of Coenzyme Q10 Supplementation on the Contrast Sensitivity Score, Biomarkers of Oxidative Stress, Inflammation, and β-Secretase Activity in the Blood and the Correlation Among these Factors in Diabetic Retinopathy Patients; Clinical Trial

**DOI:** 10.34172/apb.46420

**Published:** 2026-05-19

**Authors:** Mohammad Hossein Sharif, Fatemeh Javadian Sarraf, Ehsan Sharifipour, Masumeh Zamanlu, Parsa Gholipour, Alireza Soltanian, Siamak Shahidi, Abdolrahman Sarihi

**Affiliations:** ^1^Neurophysiology Research Center, Institute of Neurosciences and Mental Health, Hamadan University of Medical Sciences, Hamadan, Iran; ^2^Department of Neuroscience, School of Advanced Medical Sciences and Technologies, Hamadan University of Medical Sciences, Hamadan, Iran; ^3^Taban Eye Clinic, Qom, Iran; ^4^Department of Neurology, School of Medicine, Shohada-e-Tajrish Hospital, Shahid Beheshti University of Medical Sciences, Tehran, Iran; ^5^Resuscitation and Critical Care Medicine Research Center, Tabriz University of Medical Sciences, Tabriz, Iran; ^6^Modeling of Noncommunicable Diseases Research Center, Institute of Health Sciences and Technologies, Avicenna Health Research Institute, Hamadan University of Medical Sciences, Hamadan, Iran

**Keywords:** Diabetic retinopathy, Coenzyme Q10, β-secretase, Oxidative stress, Inflammation, Contrast sensitivity

## Abstract

**Introduction:**

Diabetic retinopathy (DR), the most common complication of diabetes and is recognized as the leading cause of visual impairment. Coenzyme Q10 (CoQ10) is a nonenzymatic antioxidant that restores other antioxidants, and its benefits in reducing inflammation in various disorders have been demonstrated. In this context, the primary aim of this survey was to evaluate the beneficial effects of CoQ10 on oxidative status, pro-inflammatory cytokines, β-secretase (BACE1) activity, and contrast sensitivity (CS) score in DR patients, and more importantly, the correlation of BACE1 with oxidative status and inflammatory parameters.

**Methods:**

74 DR patients (38 females and 36 males) were divided into two groups as placebo and CoQ10. The treatment process took 12 weeks. One group received 200mg cellulose acetate as a placebo, and the other group received 200 mg CoQ10. Before and after finishing the treatment process, 10ml of venous blood samples were taken from all participants to measure changes in CoQ10, Total Antioxidant Capacity (TAC), Total Oxidant Status (TOS), Glutathione (GSH), Malondialdehyde (MDA), Catalase activity (CAT), tumor necrosis factor-alpha (TNF-α), interleukin-6 (IL-6), C-reactive protein (CRP), and BACE1 activity in the serum. Moreover, the CS scores were also evaluated.

**Results:**

all the measured oxidative stress and inflammatory parameters, BACE1 activity, and also CS score were attenuated after treatment with CoQ10 in the DR patients (*P*<0.05). Moreover, after evaluating the correlation between BACE1 activity with oxidative stress and inflammatory paradigms, it was shown that there were significant correlations.

**Conclusion:**

CoQ10 supplementation increases CS score in DR patients, which can be due to a decrease in BACE1 activity. Furthermore, the reduction in BACE1 activity in DR patients was partly related to the enhancement of the antioxidant defense system and a reduction in inflammation, as there was a high correlation between BACE1 activity and these parameters.

## Introduction

 Diabetes is a chronic metabolic disorder marked by high blood glucose levels due to impaired insulin production or function, leading to disruptions in carbohydrate, lipid, and protein metabolism. It is categorized into three main types: (1) Type 1 diabetes, an autoimmune condition causing insulin deficiency; (2) Type 2 diabetes, characterized by insulin resistance and progressive beta-cell dysfunction; and (3) gestational diabetes, which emerges during pregnancy and raises the risk of future Type 2 diabetes in both mother and child. Type 1 diabetes predominantly affects younger individuals, with genetic and environmental factors contributing to its development. In contrast, Type 2 diabetes is strongly linked to modifiable risk factors such as obesity, sedentary lifestyle, poor diet, and aging. Gestational diabetes shares similar risk factors, including family history, excess weight, and advanced maternal age.^1^ The global burden of diabetes is escalating, with the International Diabetes Federation (IDF) projecting a rise from 171 million to 592 million cases by 2035.^2^ This surge poses significant challenges to healthcare systems and economies. Diabetes also induces neurochemical and structural brain changes,^3^ with diabetic retinopathy (DR) being one of its most severe complications and a leading cause of blindness in adults.^4^ DR progresses through two stages: non-proliferative (NPDR) and proliferative (PDR). NPDR involves microaneurysms, hemorrhages, and lipid deposits due to retinal capillary damage, often asymptomatic in early stages. PDR is marked by abnormal blood vessel growth, increasing the risk of vitreous hemorrhage and retinal detachment. Diabetic macular edema (DME), a consequence of fluid leakage into the macula, can occur at any DR stage and severely impairs central vision. Poor glycemic control, hypertension, and prolonged diabetes duration accelerate DR progression.^5,6^ In Type 1 diabetes, retinopathy is rare in the first 3–5 years but affects nearly all patients after 20 years. Among Type 2 diabetics, ~21% show retinal changes at diagnosis, with most developing DR over time. Studies indicate that within 15 years of diagnosis, 80% of Type 1 patients and a significant proportion of Type 2 patients develop retinopathy, with PDR peaking around 13–14 years.^7^ Mechanistically, DR involves hyperglycemia-induced pathways such as hexosamine biosynthesis, leading to abnormal protein modifications.^8^ Chronic high glucose levels promote vascular inflammation, pericyte loss, and oxidative stress, disrupting retinal blood flow. Key mediators like TNF-α, IL-1, and PKC-δ exacerbate endothelial damage, while impaired platelet-derived growth factor (PDGF) signaling contributes to cell death.^9,10^ These processes trigger microaneurysms, ischemia, and pathological neovascularization, culminating in vision loss.^11,12^

 Under normal physiological conditions, only 0.1%–5% of mitochondrial oxygen consumption generates reactive oxygen species (ROS) like superoxide, with the majority supporting metabolism; additional ROS sources include cytochrome P450, NAD(P)H oxidases, and nitric oxide synthases.^13^ While ROS are natural byproducts of cellular processes, diabetes disrupts this balance, driving excessive ROS production that overwhelms antioxidant defenses, such as glutathione, vitamin E, catalase, and superoxide dismutase (SOD), leading to oxidative stress.^14^ This stress damages DNA, proteins, and lipids, and is a central mediator of diabetic complications, including DR.^15^ In hyperglycemia, glucose autoxidation, redox imbalances, and diminished antioxidant capacity (e.g., reduced glutathione levels and SOD/catalase activity generate oxidative products that link hyperglycemia to retinal damage.^16^ The retina is uniquely vulnerable due to its high oxygen demand, polyunsaturated lipid content (prone to peroxidation), and intense metabolic activity.^17^ In DR, chronic hyperglycemia amplifies retinal oxidative stress through elevated superoxide and hydrogen peroxide levels, alongside markers like lipid peroxides and 8-OHdG (indicating DNA damage).^15^ Critically, oxidative stress not only initiates DR but also perpetuates its progression via metabolic memory, where residual ROS and unresolved molecular dysfunction sustain damage even after glucose normalization. This persistence is exacerbated by compromised retinal antioxidant defenses, reduced glutathione peroxidase, glutathione reductase,^18^ and vitamins C/E,^19^ and diminished enzymatic repair mechanisms.^20^ Furthermore, many studies illustrated that excess ROS is eliminated by specific antioxidant scavengers and balanced by mitochondria, maintaining redox,^21^ and several antioxidant scavengers such as catalase, SOD, and glutathione peroxidase (GPx) have been reported to be involved in oxidative stress during DR.^22-24^ Accumulated data from diabetic patients, diabetic animal models, and high glucose-treated cells have revealed that these antioxidants may exhibit different gene expression patterns and activity; the activity of catalase, SODs, and GPxs was reported to be low in diabetic patients, animal models, and high glucose-treated cells compared to normal controls.^22-24^ Thus, despite oxidative stress being a recognized cornerstone of DR, the precise pathways connecting it to retinal vascular leakage, neurodegeneration, and inflammation remain unclear; however, it seems that a therapeutic strategy to directly decrease ROS production and enhance expression of these antioxidants will protect the retina from oxidative stress damage during DR.

 β-Site amyloid precursor protein cleaving enzyme or β-secretase (BACE1), an aspartyl protease, catalyzes the rate-limiting step in c-amyloid (Aβ) production by cleaving amyloid precursor protein (APP).^25^ While BACE inhibitors have emerged as promising therapeutic candidates for Alzheimer’s disease (AD),^26^ recent studies highlight their dual isoforms, BACE1 and BACE2 (68% homology), as critical players in retinal pathophysiology. BACE1 is the primary isoform involved in APP processing, whereas BACE2 exhibits minimal APP-cleaving activity. However, both isoforms are abundantly expressed in the retina,^27-29^ where their dysregulation may contribute to retinal amyloid deposition, a hallmark of aging and neurodegenerative eye diseases.^29,30^ Notably, BACE1’s role extends beyond Aβ generation. It cleaves substrates such as neuregulin, interleukin-1 receptor 2, insulin receptor, and VEGFR1, influencing cell signaling, angiogenesis, and ion transport.^28,31^ This broad substrate specificity underscores its potential involvement in retinal pathologies. For example, BACE1 knockout mice develop retinal thinning, apoptosis, reduced vascular density, and lipofuscin accumulation—features reminiscent of age-related macular degeneration (AMD).^32^ Conversely, BACE1 inhibition exacerbates choroidal neovascularization (a hallmark of wet AMD) and disrupts lysosomal function, suggesting isoform-specific roles in retinal homeostasis. Intriguingly, BACE2 knockout alone, or combined BACE1/BACE2 deletion, does not amplify retinal pathology beyond BACE1 knockout, implying that BACE1 drives retinal dysfunction independently.^28^ A critical link between BACE1 and oxidative stress, a key contributor to DR and AMD, has emerged. BACE1 interacts with mitochondrial dynamics: its inhibition under oxidative stress reduces mitochondrial membrane potential, increases fragmentation, and elevates cleaved caspase-3 in retinal pigment epithelium (RPE) cells.^33^ These effects correlate with decreased mitochondrial fusion proteins (OPA1, MFN1) and increased mitophagy markers (Parkin, PINK1), suggesting BACE1 safeguards mitochondrial integrity during stress.^33^ Supporting this, oxidative stress upregulates BACE1 in retinal cells,^34^ while clinical studies associate elevated BACE levels with oxidative damage in AMD, an age-related impairment in the retina.^35,36^ The role of BACE in apoptosis remains contentious. While Aβ overproduction (linked to BACE1 activity) correlates with caspase-3 activation in optic nerve injury models,^37^ BACE2 may counteract apoptosis in some contexts.^38^ Ischemic conditions elevate BACE1 levels alongside TUNEL-positive apoptotic cells,^39^ yet Aβ’s direct apoptotic role is debated. This duality suggests a delicate balance between BACE1 and BACE2 may regulate retinal cell survival under oxidative stress, with imbalance potentially driving pathologies like DR. Finally, BACE1’s influence extends to aberrant angiogenesis—a hallmark of proliferative DR. Its cleavage of VEGFR1 and other vascular regulators positions it as a modulator of pathological neovascularization. Collectively, these findings position BACE1 at the intersection of oxidative stress, mitochondrial dysfunction, apoptosis, and angiogenesis, implicating it as a multifaceted contributor to DR pathogenesis.

 Discovered in 1957 by Frederick Crane and colleagues,^40^ coenzyme Q10 (CoQ10) is a lipid-soluble molecule characterized by a benzoquinone ring and a side chain of 10 isoprene units, earning it the alternative name ubiquinone due to its ubiquitous presence in nature. CoQ10 exists in three redox states: fully reduced ubiquinol (CoQ10H2), semiquinone (CoQ10H), and fully oxidized ubiquinone (CoQ10). Its physiological function hinges on the reversible conversion between the oxidized (CoQ10) and reduced (CoQ10H2) forms, a process critical to its roles in cellular energy production and antioxidant defense.^41^ Biosynthesis of CoQ10 occurs via three sequential steps:^42-44^ (1) synthesis of the quinoid ring from tyrosine or phenylalanine precursors, (2) generation of the 10-unit isoprenoid side chain via the mevalonate pathway, and (3) condensation of these structures to form the final molecule. Unlike vitamins, which require dietary intake, CoQ10 is endogenously synthesized, though its levels may decline with age or disease.^45^ CoQ10’s therapeutic potential stems from two key biochemical properties. First, it acts as an essential cofactor in mitochondrial oxidative phosphorylation, facilitating adenosine triphosphate (ATP) production, a function exploited clinically to support high-energy tissues like cardiac muscle. Second, its redox-active structure enables potent antioxidant activity, allowing CoQ10 to neutralize free radicals and mitigate oxidative stress. These dual roles underpin its investigational use in metabolic and degenerative disorders, including diabetes-related complications.^46,47^ Preclinical studies highlight CoQ10’s capacity to counteract diabetes-induced oxidative damage. In rodent models, supplementation reduces systemic oxidative stress,^48^ enhances insulin sensitivity,^21^ and alleviates nephropathy,^49^ peripheral neuropathy,^50^ and cerebral injury.^51^ Furthermore, CoQ10 stabilizes mitochondrial membranes, sustains ATP synthesis, and suppresses reactive oxygen species (ROS) in neurodegenerative contexts,^52^ suggesting broad cytoprotective effects. Of particular relevance to DR, CoQ10 demonstrates direct retinal benefits: it attenuates oxidative stress and ischemic injury in vitro and in vivo^53,54^ and improves endothelial dysfunction in atherosclerosis,^55^ a comorbidity often associated with diabetes. Given the central role of oxidative stress in retinal pathologies, including retinitis pigmentosa and DR, CoQ10’s antioxidant and bioenergetic properties position it as a promising therapeutic candidate for preserving retinal integrity in hyperglycemic conditions. Despite accumulating evidence on the neuroprotective and anti-inflammatory effects of CoQ10 in diabetic neuropathy and neurodegenerative disorders, its specific impact on retinal BACE1 activity, neuroinflammation, oxidative stress, and visual function in diabetic retinopathy remains extensively unexplored. Addressing this gap is critical, as these pathways are directly implicated in retinal neurovascular damage and visual dysfunction. Therefore, the present study is designed to investigate the effects of CoQ10 on BACE1 activity, associated oxidative and inflammatory markers, and visual functions in DR patients. By elucidating these mechanisms, our research not only provides novel insights into the potential therapeutic role of CoQ10 in DR but also lays the groundwork for translational strategies aimed at preserving retinal structure and visual function, such as contrast sensitivity, in diabetic neuropathic patients. As an initial exploratory study, our work focuses primarily on serum biomarkers and limited functional outcomes, with the aim of providing foundational evidence that may justify more extensive, mechanistic, and long-term investigations in future research.

## Materials and Methods

###  Study Design and Participants

**Figure 1:**
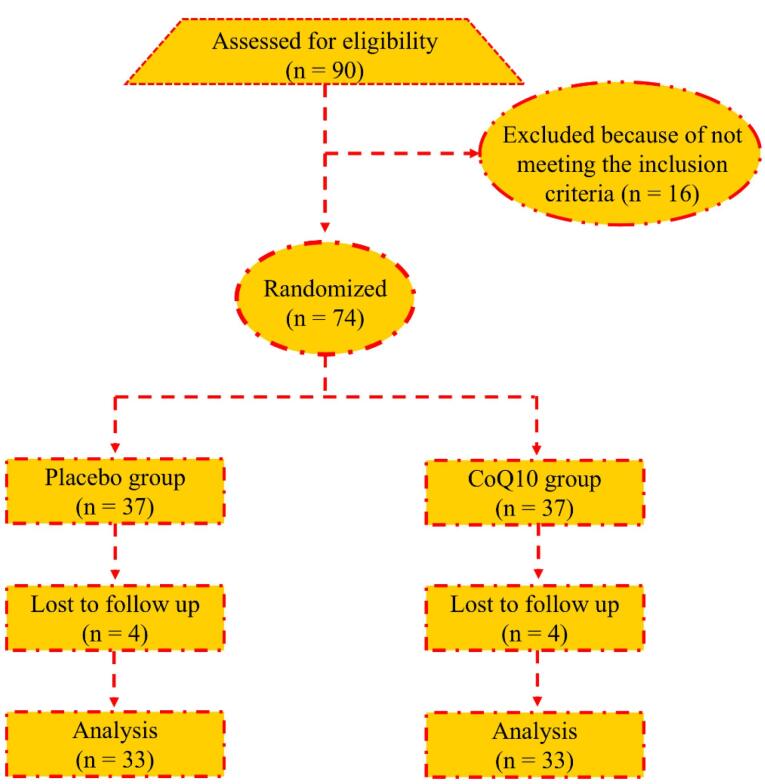
Summary of patient flow diagram

 This randomized, double-blind, placebo-controlled trial was conducted at the Taban clinic, a tertiary eye care center in Qom, Iran. Seventy-four patients (38 females and 36 males) diagnosed with DR, aged between 47 and 75, with a history of diabetes lasting from 2 to 20 years, enrolled in this trial and were divided into two groups: one group receiving Q10 treatment and the other receiving a placebo. However, during the study, patients who participated for less than 70% of the time (in terms of consuming the treatment) were removed from the analysis. At the end, data from 33 patients in each group were analyzed (Figure 1). The study protocol was approved by the ethics committee of the Hamadan University of Medical Sciences (UMSHA, code: IR.UMSHA.REC.1400.969), and informed consent forms were signed by all participants before the intervention. The process of the study took place from January to November 2024. The minimum sample size was calculated to be 30 for each group, using a statistical power of 80% and a confidence interval of 95%, according to the other studies.^56^ All the participants were Iranian people who had not consumed antioxidant and vitamin supplements for at least 3 months before this study, and the diagnosis of diabetes was based on the World Health Organization criteria. Moreover, the mental health of patients was assessed using the Mini-Mental State Examination (MMSE), and after taking a score of over 28 (MMSE > 28) out of 30 on this examination, they became eligible to start the survey. The exclusion criteria were (1) taking an score of under 28 (MMSE < 28) out of 30 in the MMSE (2) uncontrolled systolic blood pressure exceeding 150 mmHg; (3) uncontrolled blood glucose (fasting blood glucose exceeding 200 mg/dL); (4) uncontrolled blood cholesterol levels; (5) diagnosed with vision-impairing ocular disorders, such as glaucoma or other retinal disorders; (6) diagnosed with other central nervous system pathologies, including stroke, tumors, trauma, Alzheimer’s disease, Parkinson’s disease, and related disorders; (7) chronic thyroid, gastrointestinal, liver, kidney, and blood diseases; (8) smoking; (9) alcoholism; (10) pregnancy or lactation; (11) using anticoagulants, diuretics, and β blockers; (12) hormone therapy; (13) insulin therapy; (14) changes in type and dosage of glucose and lipid-lowering drugs; (15) changes in diet and physical activity levels during the intervention; and (16) unwillingness to cooperate in the study. To increase compliance rates, all participants received short messages on their cell phones every day to remind them about taking the intervention. In addition to evaluating the compliance, we counted the remaining supplements. Participants were requested to keep their habitual diet and routine levels of physical activity and not to take any medication that could affect the outcome throughout the study period. Finally, it should be noted that no side effects were observed among the patients consuming the placebo and CoQ10. Only three patients reported experiencing minor side effects because they took the treatment before lunch. They were advised to use it after lunch, which eliminated the side effects.

###  Retinopathy Diagnosis Procedure

 In this study, Diabetic patients were invited to the Taban clinic and underwent the retinopathy diagnosis procedure. Retinopathy was diagnosed using the scanning laser angiography method. For this method, a two-dimensional (2D) optical coherence tomography (OCT) angiography scan (HRA Spectralis, Heidelberg, Germany- laser source angiography- 486 nm) was used. The exact process by which the retinopathy was diagnosed among diabetic patients is explained in the previous works.^57^ Then, after reaching the images, the specialists independently identified and graded them. The graders were masked to the background information of the patients in each image. Only images with consistent results from the graders of the existence and the presence or absence of hyporeflective areas were used for the analyses.

###  Medication Protocol

 The patients were randomly assigned into two separate groups, one of which received an oral dosage of 200 mg of CoQ10 supplement capsule daily (Jalinous, Iran), and the other group was administered one capsule including 200 mg cellulose acetate (Sigma-Aldrich, USA) as a placebo daily for 12 weeks.^58^ As a double-blinded study, patients and researchers did not have any clinical involvement in the trial. The supplement and placebo capsules were identical in appearance and packed in the same packs. Patients were called to remind them about the consumption of the capsules every 2 weeks.

###  Demographic and Anthropometric Measurements

 Demographic and anthropometric information of the participants, including age, duration of suffering from diabetes, body mass index (BMI), systolic blood pressure (SBP), and diastolic blood pressure (DBP), was measured at the beginning of the study.

###  Contrast Sensitivity Measurement Using Freiburg Visual Acuity and Contrast Test (FrACT)

 For the assessment, we employed the Freiburg Visual Acuity and Contrast Test 10 (FrACT10), version 1.0.5 (1). Measurements were performed at a fixed viewing distance of 50 cm, using appropriate near correction when needed, with a Landolt C optotype corresponding to a diameter of 125 arcminutes. To facilitate better performance in elderly participants, the standard set of eight possible gap orientations was reduced to four response options, resulting in a 25% chance level. Threshold estimation in FrACT is based on the PEST (Best Parameter Estimation by Sequential Testing) adaptive algorithm. To prevent unintentional adjustments by the subjects, the examiner manually recorded all responses. Each testing session consisted of 24 trials with Landolt C stimuli, as recommended by the software guidelines, and three initial simple items were added to improve patient confidence. Stimuli were displayed for up to 60 seconds per trial. The test was run on an HP EliteBook 2760p with a 1280 × 800-pixel display operating at a refresh rate of 39.984 Hz. The device was powered on at least 30 minutes before testing to minimize fluctuations in screen luminance. During every session, participants were instructed to maintain a perpendicular viewing angle to the display, as variations in gaze angle can influence perceived contrast and illumination.^59^ Contrast sensitivity was expressed in logarithmic units (logCS), consistent with visual science standards, and to provide a linear scale suitable for statistical analysis.

###  Blood Samples

 After 10 h of fasting, 10 ml of venous blood samples were taken from all the participants and were centrifuged for 10 min at 3000 rpm, and serum samples were separated and stored at −70°C for assessing biochemical parameters.

###  Biochemical Assessment

 The BACE1 activity was evaluated using a fluorometric ELISA kit (ZellBio GmbH, Germany), and the measurements were performed according to the company’s instructions. The oxidative stress factors of total antioxidant capacity, total oxidant status, catalase activity, Malondialdehyde, and glutathione were bought from Kia Zist assay kit (Iran), and they are measured using the instructions of this company. Inflammatory parameters of Tumor necrosis factor-α, Interleukin 6, and C-reactive protein were purchased from Karmania Pars Gene company (Iran) and measured using the method described in the brochure. All the biochemical evaluations were done at both baseline and at the end of the study.

###  Statistical Analysis

 PRISM software version 10 (GraphPad Software, San Diego, CA, USA) was used for data analysis. mean ± Standard Deviation was used for presenting quantitative data, while qualitative data were expressed regarding frequency. The Shapiro-Wilk test was used to assess the normality of the data distribution, and a paired t-test for within-group differences and an independent t-test for between-group differences were used. In addition, Pearson’s correlation was used to evaluate the potential correlation among the parameters. significance level of less than 0.05 (*P* < 0.05) was considered statistically significant.

## Results

 The results of the Shapiro-Wilk and independent t-tests on the pre-test data showed that the data distribution in both the CoQ10 and the Placebo groups was normal, and in both groups, before the intervention of independent variables, there were no significant differences in all dependent variables. In simpler terms, there were no significant differences between the two groups in the baseline conditions of all the measured parameters (*P* > 0.05).

###  A Summary of Demographic and Anthropometric Data of the Subjects

**Figure 2:**
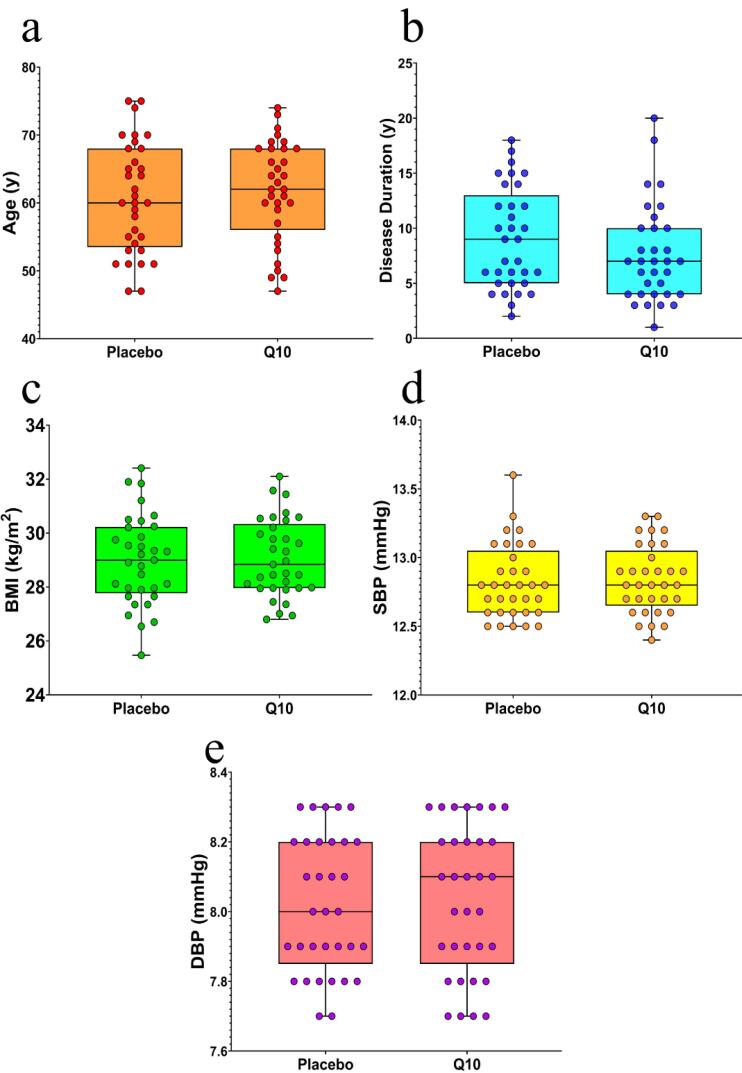
Comparison of demographic and anthropometric data of the subjects before the intervention. BMI: Body Mass Index; SBP: Systolic Blood Pressure; DBP: Diastolic Blood Pressure. Each bar represents the mean ± SD of 33 samples per group

 At the start of the study, the baseline characteristics, including age, duration of the disease, BMI, systolic blood pressure (SBP), and diastolic blood pressure (DBP), were measured. In this study, 66 patients struggling with DR were included, and these individuals were present until the end of the research. Based on the obtained results, the mean age of individuals in the placebo group was (60.82 ± 8.145 years), and in the CoQ10 group, it was (61.7 ± 7.435 years). The mean disease duration in the Placebo group was (9.061 ± 4.582 years), and in the CoQ10 group, it was (7.606 ± 4.43 years). Additionally, the mean of Body Mass Index (BMI) in the placebo group was (28.99 ± 1.654 kg/m^2^), and in the CoQ10 group, it was (29.07 ± 1.429 kg/m^2^). Finally, after evaluating both SBP and DBP, it was shown that the mean of SBP and DBP in the placebo group were (12.82 ± 0.2693 mmHg) and (8.018 ± 0.1944 mmHg), respectively, and the mean of these parameters in the CoQ10 group were (12.84 ± 0.2461 mmHg) and (8.033 ± 0.2102 mmHg), respectively.Table 1 and Figure 2 indicate the mean and standard deviation of the mentioned variables in the present study before the treatment program begins, and compare the two groups in each criterion of the study.

**Table 1:** Comparison of demographic and anthropometric data of the subjects before the intervention. BMI: Body Mass Index; SBP: Systolic Blood Pressure; DBP: Diastolic Blood Pressure.

**variables**	**Placebo** **(mean±SD)** **(n=33)**	**Q10** **(mean±SD)** **(n=33)**	***P*** **-Value**
Age (y)	60.82 ± 8.145	61.70 ± 7.435	0.6487
Disease Duration (y)	9.061 ± 4.582	7.606 ± 4.430	0.1945
BMI (kg/m2)	28.99 ± 1.654	29.07 ± 1.429	0.8422
SBP (mmHg)	12.82 ± 0.2693	12.84 ± 0.2461	0.8122
DBP (mmHg)	8.018 ± 0.1944	8.033 ± 0.2102	0.7621

###  The Effect of CoQ10 Treatment on the CoQ10 Levels in the Serum of Patients with DR

**Figure 3:**
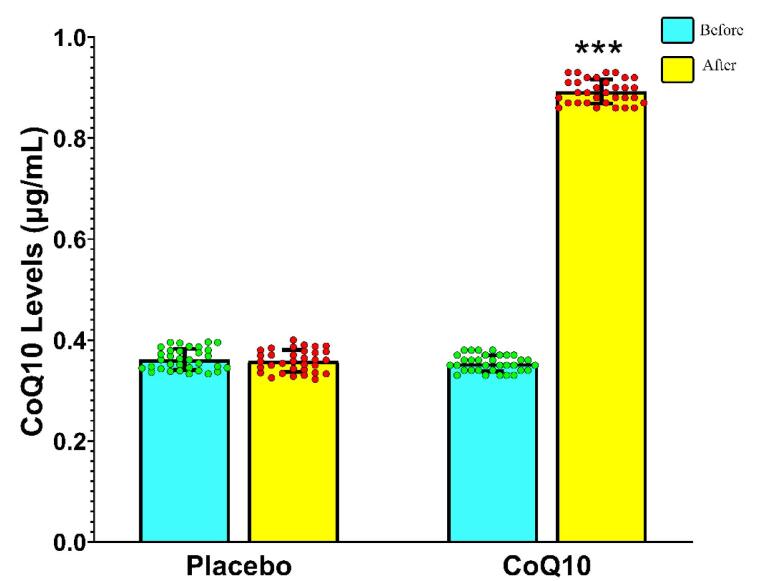
Changes in the CoQ10 levels in the serum before and after intervention in the two groups. CoQ10: Coenzyme Q10. Comparisons were performed using paired t-tests within groups and unpaired t-tests between groups. Each bar represents the mean ± SD of 33 samples per group. ***: *P* < 0.001 indicates the significance of the post-test values compared to the pre-test values

 The results of this part of the present study, using a paired t-test, illustrated that after 12 weeks of CoQ10 treatment, a notable difference was seen in the levels of CoQ10 among the DR patients (*P* < 0.001), where the mean of serum CoQ10 concentrations increased from 0.3536 ± 0.01617 to 0.8924 ± 0.02398. Moreover, between-group comparisons using an unpaired t-test showed that there was a significant difference between the CoQ10 and the placebo groups in the levels of CoQ10 in their blood after 12 weeks (*P* < 0.001) (Table 2 and Figure 3).

**Table 2:** Comparison of the biochemical parameters of the subjects before and after the intervention. CoQ10: Coenzyme Q10; TAC: Total Antioxidant Capacity; TOS: Total Oxidant Status; GSH: Glutathione; MDA: Malondialdehyde; CAT: Catalase Activity; TNF-α: Tumor Necrosis Factor-α; IL-6: Interleukin-6; CRP: C-reactive protein; BACE1: β-secretase; CS: Contrast Sensitivity.

**variables**	**Placebo (n=33)**			**Q10 (n=33)**		
	**pre-test** **(mean±SD) **	**post-test** **(mean±SD)**	**Difference** **(mean±SD)**	**pre-test** **(mean±SD) **	**post-test** **(mean±SD) **	**Difference** **(mean±SD)**
CoQ10 (μg/mL)	0.3615 ± 0.02086	0.3585 ± 0.02175	-0.002909 ± 0.01030	0.3536 ± 0.1617	0.8924 ± 0.02398	0.5388 ± 0.02643
TAC (mmol/L)	0.3224 ± 0.05879	0.3288 ± 0.07008	0.006364 ± 0.08975	0.3255 ± 0.07045	0.4012 ± 0.06604	0.07576 ± 0.1111
TOS (mmol/L)	7.199 ± 1.707	7.159 ± 1.666	-0.04061 ± 1.281	7.181 ± 2.025	6.269 ± 1.634	-0.9115 ± 1.464
GSH (mg/dL)	2.018 ± 0.1092	2.045 ± 0.1201	0.2667 ± 0.158	2.007 ± 0.09184	2.129 ± 0.1084	0.1227 ± 0.1363
MDA (μmol/L)	7.259 ± 1.468	7.108 ± 1.328	-0.1512 ± 0.8093	7.276 ± 1.392	6.105 ± 1.274	-1.171 ± 1.572
CAT (KU)	2.056 ± 0.3463	2.108 ± 0.3375	0.05182 ± 0.2418	1.981 ± 0.3141	2.423 ± 0.4137	0.4418 ± 0.2788
TNF-α (pg/mL)	20.87 ± 3.184	21.11 ± 2.92	0.2424 ± 1.606	21 ± 2.393	17.76 ± 2.618	-3.236 ± 1.453
IL-6 (pg/mL)	5.891 ± 1.481	5.9 ± 1.498	0.009091 ± 0.4333	5.888 ± 1.548	4.667 ± 1.27	-1.221 ± 0.6886
CRP (mg/L)	3.145 ± 2.451	3.176 ± 2.406	0.03097 ± 1.073	4.039 ± 3.501	2.937 ± 2.039	-1.102 ± 1.82
BACE1 (kU/L)	43.62 ± 6.228	44.43 ± 5.876	0.8148 ± 5.959	43.91 ± 5.796	36.3 ± 9.949	-7.607 ± 12.18
CS (LogCS)	1.135 ± 0.4176	1.137 ± 0.4422	0.001818 ± 0.2534	1.127 ± 0.4996	1.241 ± 0.3813	0.1142 ± 0.2815

###  The Effect of CoQ10 Treatment on the Oxidative Stress Resulting from DR

**Figure 4:**
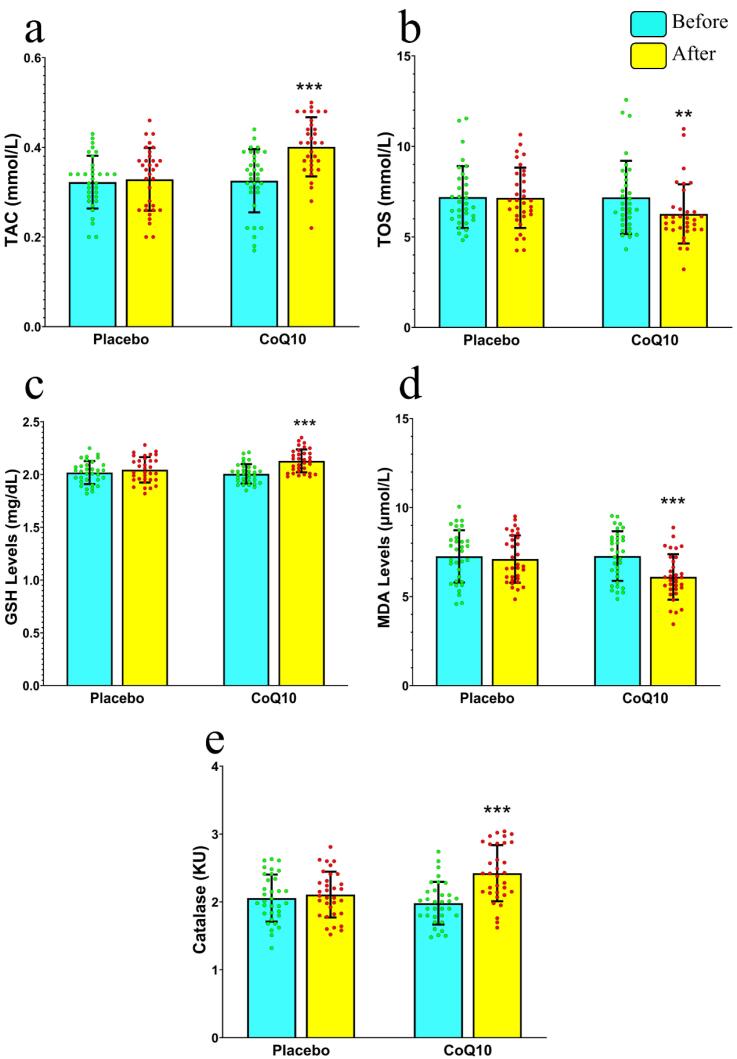
Changes in the oxidative stress status in the serum before the intervention in the two groups. a) TAC, b) TOS, c) GSH, d) MDA, e) CAT. TAC: Total Antioxidant Capacity; TOS: Total Oxidant Status; GSH: Glutathione; MDA: Malondialdehyde; CAT: Catalase Activity; KU: Katal Unit. Comparisons were performed using paired t-tests within groups and unpaired t-tests between groups. Each bar represents the mean ± SD of 33 samples per group. **: *P* < 0.01, ***: *P* < 0.001 indicate the significance of the post-test values compared to the pre-test values

 After analyzing the results of this section using paired t-test, it was shown that after 12 weeks of intervention, CoQ10 supplementation significantly increased the mean of the levels of total antioxidant capacity (TAC) (from 0.3255 ± 0.07045 to 0.4012 ± 0.06604; *P* < 0.001), Glutathione (GSH) (from 2.007 ± 0.09184 to 2.129 ± 0.1084; *P* < 0.001), and Catalase activity (CAT) (from 1.981 ± 0.3141 to 2.423 ± 0.4137; *P* < 0.001), and also it decreased the levels of total oxidant status (TOS) (from 7.181 ± 2.025 to 6.269 ± 1.634; *P* < 0.01) and Malondialdehyde (MDA) (from 7.276 ± 1.392 to 6.105 ± 1.274; *P* < 0.001). In addition, an unpaired t-test was utilized for intra-group comparison and indicated that the differences of pre- and post-test variables between the two groups were significant in TAC (*P* < 0.05), TOS (*P* < 0.05), GSH (*P* < 0.05), MDA (*P* < 0.01), and CAT (*P* < 0.001) (Table 2 and Figure 4).

###  The Effect of CoQ10 Treatment on the Inflammation Resulting from DR

**Figure 5:**
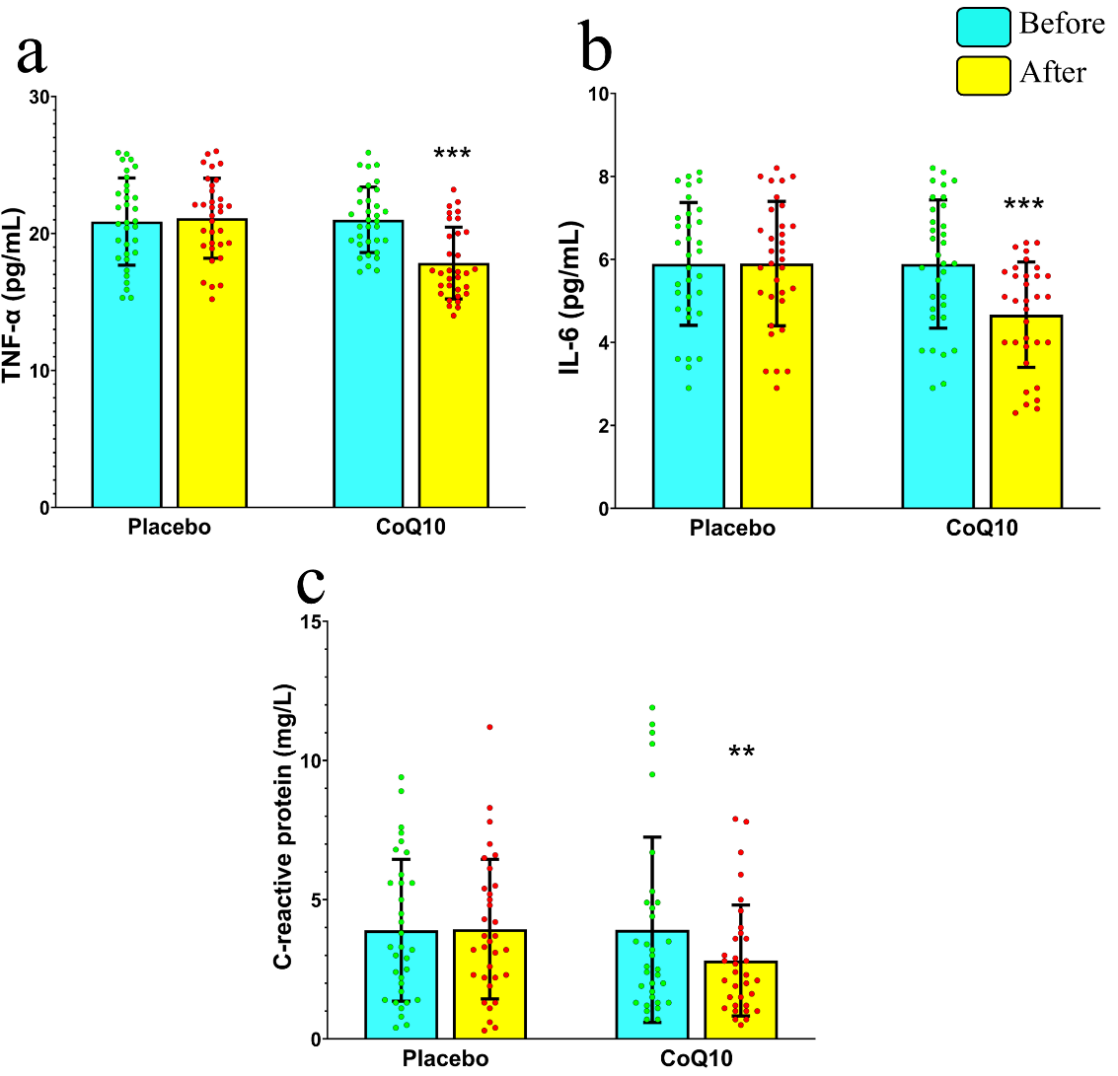
Changes in inflammation status in the serum before and after intervention in the two groups. a) TNF-α, b) IL-6, c) CRP. TNF-α: Tumor Necrosis Factor-α; IL-6: Interleukin-6; CRP: C-reactive protein. Comparisons were performed using paired t-tests within groups and unpaired t-tests between groups. Each bar represents the mean ± SD of 33 samples per group. **: *P* < 0.01, ***: *P* < 0.001 indicate the significance of the post-test values compared to the pre-test values

 After analyzing inflammatory cytokines and C-reactive protein (CRP) using a paired t-test, it was clear that there was a significant reduction in all these inflammatory parameters. To be more precise, the mean of Tumor Necrosis Factor-α (TNF-α) in the CoQ10 group decreased from 21 ± 2.393 to 17.76 ± 2.618 (*P* < 0.001), Interleukin-6 (IL-6) declined from 5.888 ± 1.548 to 4.667 ± 1.27 (*P* < 0.001), and finally, CRP depressed from 3.918 ± 3.329 to 2.816 ± 1.993 (*P* < 0.01). Moreover, unpaired t-test analysis illustrated that there was a significant difference between the two groups in the differences of pre- and post-test results in all the inflammatory parameters, including TNF-α (*P* < 0.001), IL-6 (*P* < 0.001), and C-reactive protein (*P* < 0.01) (Table 2 and Figure 5).

###  The Effect of CoQ10 Treatment on the β-Secretase Activity in the Serum of Patients with DR

**Figure 6:**
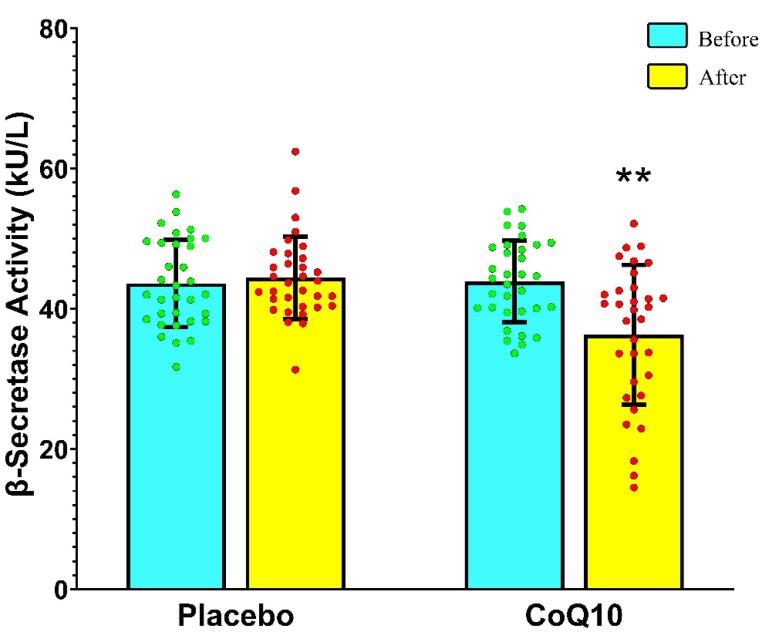
Changes in BACE1 activity in the serum before and after intervention in the two groups. kU/L: kilo-Units per liter. Comparisons were performed using paired t-tests within groups and unpaired t-tests between groups. Each bar represents the mean ± SD of 33 samples per group. **: *P* < 0.01 indicates the significance of the post-test values compared to the pre-test values

 BACE1 activity was analyzed before and after the consumption of CoQ10, and the results of the paired t-test demonstrated that there was a significant reduction in the mean activity of BACE1 after the consumption of CoQ10, where it decreased from 43.91 ± 5.796 to 36.3 ± 9.949 (*P* < 0.01). Furthermore, concerning the results of the unpaired t-test on intra-group changes, there was a significant difference between the differences of pre- and post-test variables among the two groups (*P* < 0.001) (Table 2 and Figure 6).

###  The Effect of CoQ10 Treatment on the Contrast Sensitivity of Patients with DR

**Figure 7:**
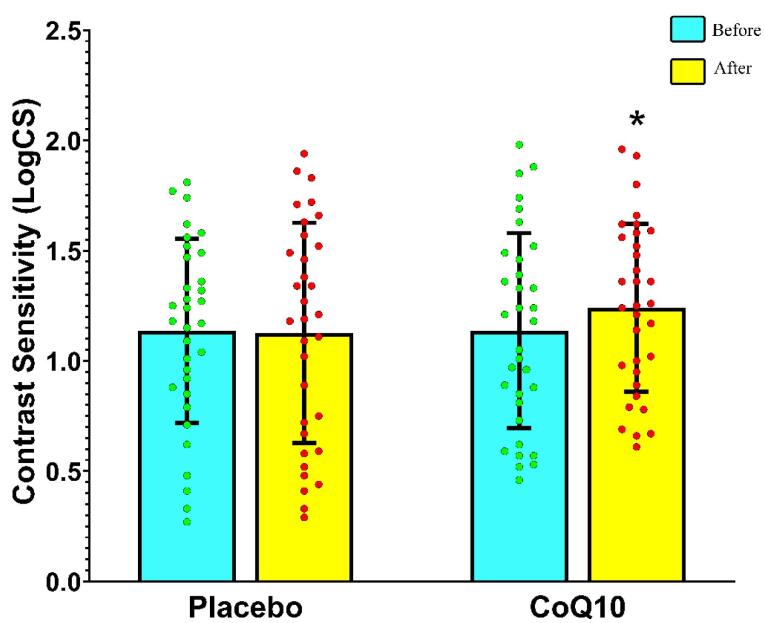
Changes in CS score before and after intervention in the two groups. Comparisons were performed using paired t-tests within groups and unpaired t-tests between groups. Each bar represents the mean ± SD of 33 samples per group. *: *P* < 0.05 indicates the significance of the post-test values compared to the pre-test values

 CS was analyzed before and after the consumption of CoQ10, and the results of the paired t-test demonstrated that there was a significant reduction in the mean score of CS after the consumption of CoQ10, where it increased from 1.127 ± 0.4996 to 1.241 ± 0.3813 (*P* < 0.05). Furthermore, concerning the results of the unpaired t-test on intra-group changes, there was no significant difference between the differences of pre- and post-test variables among the two groups (*P* > 0.05) (Table 2 and Figure 7).

###  Pearson’s Correlation Between β-secretase Activity and CoQ10 Levels, Oxidative Stress, and Inflammation in the Serum of Patients with DR

**Figure 8:**
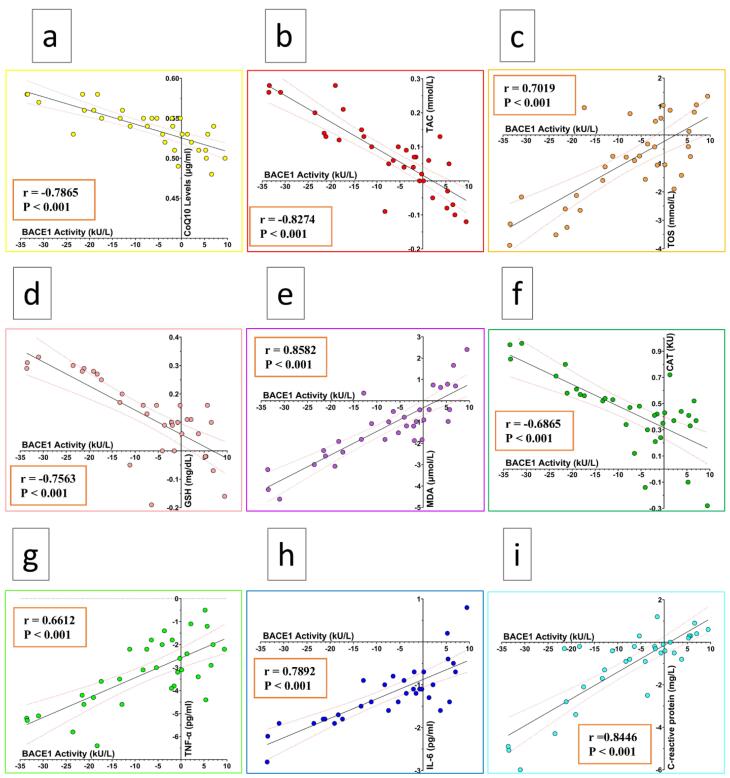
Pearson’s correlation analysis was performed to evaluate the relationships between coQ10 levels (a), oxidative stress (b-f), and inflammation (g-i) with β-secretase activity in the serum after treatment with coQ10

 The negative correlations between the levels of coenzyme Q10 in the serum and activity of BACE1 (r = -0.7865, *P* < 0.001) were observed (Figure 8a) in the DR patients after consumption of CoQ10. Specifically, the data indicate that the decreased BACE1 activity in the DR patients was associated with higher levels of coenzyme Q10 in patients’ serum after consumption of CoQ10.

 Significant inverse correlations were observed between oxidative biomarkers of TAC (Figure 8b), GSH (Figure 8d), and CAT (Figure 8f), and BACE1 activity, with respective r-values of -0.8274, -0.7563, and -0.6865, and *P*-values of < 0.001. Moreover, there was a direct correlation between oxidative biomarkers of TOS (Figure 8c), MDA (Figure 8e), and BACE1 activity, with r-values of 0.7019 and 0.8582, respectively, and *P*-values of < 0.001. These outcomes illustrate that the increase in antioxidant defense seen in DR patients using treatment with CoQ10 may be strongly tied to reduce BACE1 activity within the serum.

 Finally, it could be seen that there were positive correlations between the inflammatory biomarkers in the serum of DR patients and the level of TNF-α (r = 0.6612, *P* < 0.001, Figure 8g), IL-6 (r = 0.7892, *P* < 0.001, Figure 8h), and CRP (r = 0.8446, *P* < 0.001, Figure 8i) in this part of the body after undergoing CoQ10 treatment. Therefore, these data suggest that the decreased activity of BACE1 in the serum of DR patients was related to decreased inflammation through the consumption of CoQ10 (the P and r-values related to correlation analysis are also located in Figure 8).

## Discussion

 To the best of our knowledge, this clinical trial was the first study on human subjects to determine the effects of CoQ10 supplementation on the activity of serum BACE1 in DR patients. Based on the current research, we observed that oral administration of 200 mg/day of CoQ10 in patients suffering from DR for 12 weeks could lead to a significant increase in serum CoQ10, TAC, and GSH levels, and CAT activity; and a significant decrease in TOS, MDA, TNF-α, IL-6, and CRP levels, and BACE1 activity after 12 weeks. These therapeutic effects further cause an enhancement in CS function in the retina of these patients. Additionally, there was a significant correlation between the changes in the BACE1 activity and CoQ10 levels in the serum, as well as BACE1 activity and both inflammatory cytokines and oxidative stress biomarkers in the serum. In this study, we have demonstrated the antioxidant and anti-inflammatory effects of CoQ10 supplementation after 12 weeks, accompanied by a reduction in BACE1 activity in DR patients. This effect was confirmed by clinical improvement in CS function.

 Growing evidence indicates that BACE1 is not merely a downstream biochemical marker but an active driver of retinal neurodegeneration in DR. A substantial body of research has established oxidative stress as a central mechanism in the development and progression of diabetic complications, including DR.^60^ Chronic hyperglycemia markedly increases intracellular ROS production through several biochemical pathways, such as accelerated glucose metabolism, glucose auto-oxidation, and enhanced formation of advanced glycation end products (AGEs).^60,61^ When AGEs interact with their receptors (RAGE), they further amplify ROS generation, leading to an imbalance between pro-oxidant production and antioxidant defense systems. This redox disequilibrium results in oxidative damage to essential biomolecules, including DNA, proteins, lipids, and carbohydrates, ultimately compromising cellular integrity and function.^62^ In retina, prolonged hyperglycemia exacerbates oxidative damage by promoting ROS accumulation, lipid peroxidation, and mitochondrial impairment, all of which contribute to bioenergetic failure and apoptosis.^61^ Hyperglycemia also accelerates non-enzymatic glycation, with AGE–RAGE engagement activating NADPH oxidase and further increasing ROS formation.^60^ Another major source of oxidative stress involves protein kinase C (PKC) activation driven by diabetes-induced diacylglycerol accumulation, which additionally stimulates NADPH oxidase–dependent ROS production.^63^ The retina is uniquely vulnerable to oxidative injury because of its exceptionally high oxygen consumption, elevated glucose oxidation rate, and high content of polyunsaturated fatty acids, making it one of the most metabolically active tissues in the body.^17^ Under these conditions, oxidative stress directly upregulates BACE1 gene expression and stabilizes its protein product via redox-sensitive pathways, particularly the JNK (C-Jun N-terminal kinases) signaling cascade and the PERK (protein kinase RNA-like ER kinase)–eIF2α (eukaryotic initiation factor 2 alpha) translational control pathway.^64^ Excess ROS activates upstream MAP kinase kinases (MKK4/7), which phosphorylate and activate JNK. Activated JNK then translocates to the nucleus, where it phosphorylates transcription factors such as c-Jun and ATF-2. These transcription factors bind to AP-1 response elements in the BACE1 promoter, enhancing its transcription. As a result, oxidative stress triggers a sustained increase in BACE1 mRNA and protein. In retinal cells, JNK activation contributes to mitochondrial dysfunction, apoptosis of retinal ganglion cells, microglial activation, and increased production of inflammatory mediators such as TNF-α and IL-6, all of which are factors known to intensify BACE1 upregulation. Thus, the JNK pathway forms a direct mechanistic link between oxidative injury, inflammation, and BACE1-mediated amyloidogenic processing.^65^

 The PERK–eIF2α pathway is a major arm of the unfolded protein response (UPR), activated under conditions of endoplasmic reticulum (ER) stress, which is common in DR due to chronic hyperglycemia, oxidative damage, and accumulation of misfolded proteins. When ER stress is triggered, PERK phosphorylates eIF2α. Phosphorylated eIF2α globally reduces protein synthesis to decrease ER burden; however, it paradoxically increases translation of certain stress-responsive mRNAs, including BACE1. This occurs because the BACE1 mRNA contains upstream open reading frames (uORFs) that enhance its translation under conditions of eIF2α phosphorylation.^64^

 Hyperglycemia-induced ROS and lipid peroxidation also impair mitochondrial respiration, generating intracellular stress signals that further enhance BACE1 enzymatic activity and promote amyloidogenic processing. In this biochemical context, CoQ10 emerges as a promising therapeutic molecule. Numerous clinical studies demonstrate its capacity to enhance endogenous antioxidant defenses. For example, Lee et al.^66^ and Liu et al.^67^ reported that 300 mg/day of CoQ10 for 12 weeks significantly increased catalase (CAT) activity in patients with coronary artery disease and hepatocellular carcinoma, respectively. Similarly, Díaz-Castro et al. found that 100 mg/day of CoQ10 for 12 weeks elevated total antioxidant capacity (TAC) in individuals with varicocele,^68^ CoQ10 appears to reduce free radical production through multiple mechanisms, including enhancement of CAT and TAC, regeneration of vitamin E via tocopherol reduction, and direct scavenging of oxygen and lipid-derived radicals.^56^ Additionally, previous study demonstrated that 100 mg/day CoQ10 for 8 weeks improved insulin metabolism markers in metabolic syndrome patients, while 200 mg/day for 12 weeks enhanced glycemic control in diabetic subjects.^56,69^ Hodgson et al.^70^ similarly reported improvements in glucose metabolism with 200 mg/day CoQ10. Through its glycemic and antioxidant benefits, CoQ10 may mitigate oxidative injury and thereby inhibit DR progression. CoQ10 may also regulate insulin metabolism by modulating insulin and adiponectin receptor signaling, tyrosine kinase activity, PI3K, and glucose transporter expression,^71^ further supporting its relevance in the metabolic dysregulation central to DR pathophysiology. In this study, we have confirmed the role of oxidative stress and its relation with retinal function in DR patients before and after CoQ10 supplement for 12 weeks.

 Simultaneously, chronic inflammation, characterized by elevated TNF-α, IL-6, and activation of NF-κB, further amplifies BACE1 expression by inducing ER stress and promoting sustained microglial activation.^72^ DR arises from a complex interplay among endothelial cells, pericytes, immune cells, and glial elements within the retinal microvasculature. Mitochondrial oxidative stress acts as an early trigger for inflammatory signaling in capillary endothelial cells, thereby accelerating vascular dysfunction and initiating downstream inflammatory cascades.^73^ In metabolically stressed tissues, macrophages orchestrate inflammatory responses,^74^ while microglia, the specialized macrophages of the retina and central nervous system, serve as key regulators of neuronal integrity through their phagocytic and surveillance functions. Persistent microglial activation in response to macrophage-derived signals perpetuates neuroinflammation^75^ by continuously releasing pro-inflammatory mediators, including ROS, that inflict collateral damage on surrounding retinal structures. In this study, we have confirmed the role of inflammation and its relation with retinal function in DR patients before and after CoQ10 supplement for 12 weeks.

 As previously noted, prolonged hyperglycemia enhances AGE accumulation, worsening endothelial injury and amplifying vascular inflammation. Retinal vascular inflammation is a central pathogenic driver of DR progression,^76^ closely associated with the upregulation of inflammatory genes and age-related microglial alterations within retinal tissue.^77^ Clinically, DR often begins with microaneurysms and intraretinal hemorrhages,^78^ later advancing to retinal ganglion cell (RGC) degeneration.^79^ Although neuronal loss alone does not immediately result in vision impairment, advanced DR is characterized by pathological neovascularization, fibrovascular scarring, tractional retinal detachment, and macular edema.^78^ The observation that neuronal dysfunction and glial activation frequently arise before overt vascular damage supports the view that DR also involves a neuropathic component,^79^ highlighting the dynamic crosstalk between neurons, microglia, and vascular cells. Endothelial injury can, in turn, activate microglia, demonstrating that inflammation is intricately involved in both vascular and neuronal degenerative pathways.

 Inflammation itself is initially a protective response to injury or infection, driven by cytokine-mediated vascular permeability changes, recruitment of immune cells, and activation of resident phagocytes. Macrophages secrete TNF-α, IL-1β, and prostaglandins, while infiltrating leukocytes can inadvertently exacerbate tissue injury through the release of ROS and proteolytic enzymes.^80^ When dysregulated, these mechanisms promote leukocyte accumulation, chronic inflammation, and pathological angiogenesis, all of which contribute to progressive retinal damage. Hyperglycemia stimulates inflammatory cytokine production in retinal glial cells, and growing evidence suggests that glial reactivity is a consequence of glucose-induced increases in vascular permeability and breakdown of the blood–retinal barrier.^81^ Pro-inflammatory mediators, including VEGF and nitric oxide, further amplify this cycle of inflammation, deepening vascular leakage, oxidative stress, and neuronal injury.^82^ In its chronic form, DR becomes characterized by persistent glial activation, continuous cytokine release, and ultimately irreversible cellular damage.

 CoQ10’s antioxidant properties help protect cells, particularly those involved in innate and adaptive immune responses, by neutralizing ROS and stabilizing redox homeostasis. In addition to its antioxidant actions, increasing evidence indicates that CoQ10 exerts direct and indirect anti-inflammatory effects, including modulation of IL-1 and TNF-α expression in patients with DR.^83^ Supplementation with CoQ10 has been shown to reduce CRP, IL-6, and TNF-α levels, supporting its role in redox-dependent anti-apoptotic and immunomodulatory pathways. Although the full anti-inflammatory mechanism of CoQ10 has not been fully elucidated, one proposed pathway involves suppression of NF-κB activity, potentially through the scavenging of reactive species that trigger NF-κB activation in monocytes.^84^ Together, these mechanisms suggest that CoQ10 may mitigate the self-amplifying cycle of oxidative stress, inflammation, and cellular injury characteristic of DR.

 In addition, chronic hyperglycemia increases the formation of ROS, which attack nuclear and mitochondrial DNA. As a result, oxidized DNA lesions such as 8-hydroxy-2’-deoxyguanosine (8-OHdG) accumulate; 8-OHdG is widely accepted as a biomarker of oxidative DNA damage.^62^ In retinal tissue from diabetic animals, increased 8-OHdG content and mitochondrial DNA (mtDNA) damage have been documented, along with impaired respiratory-chain function in mitochondria. These DNA lesions compromise mtDNA-encoded subunits of the electron transport chain (ETC), which in turn exacerbates mitochondrial dysfunction and further ROS generation, creating a self-propagating cycle of oxidative stress and DNA damage.^85^ Meanwhile, oxidative stress can upregulate the expression and activity of BACE1. In vivo experiments in rat retina have shown that mitochondrial respiratory inhibition or oxidant treatment increases BACE1 protein level, BACE activity, and accumulation of APP-derived β-CTF and Aβ. Similarly, in neuronal cell culture models, high glucose induces ROS and upregulates BACE1 and Aβ generation, an effect reversed by antioxidants.^86^ Moreover, oxidative signals such as hydrogen peroxide (H₂O₂) are able to activate BACE1 transcription. In the context of diabetes, glucose-induced ROS and downstream redox signaling (e.g., via HIF-1α) may therefore contribute to enhanced BACE1 expression and amyloidogenic processing.^86^ In retinal cells, such a mechanism can have pathologic consequences: oxidative DNA damage (e.g. 8-OHdG accumulation, mtDNA instability) may impair mitochondrial function and provoke neuronal apoptosis, as observed in animal diabetic retinopathy models. If BACE1 is concomitantly upregulated, whether directly by oxidative stress or indirectly via redox-sensitive transcriptional pathways, then increased APP β-cleavage and Aβ production could further amplify oxidative stress and DNA damage, establishing a vicious cycle of neurodegeneration. Indeed, the concept that oxidative stress and Aβ/BACE1 reinforce each other is well established in neurodegenerative contexts.^87^

 Mitochondrial impairment represents a key mechanistic link between metabolic stress and elevated BACE1 activity in DR. Chronic hyperglycemia disrupts the mitochondrial electron transport chain—particularly complexes I and III, resulting in excessive mitochondrial ROS formation, loss of membrane potential, and reduced ATP synthesis, all of which have been well documented in diabetic retinal tissues.^21^ Oxidative mitochondrial stress activates the PKR-dependent integrated stress response, leading to enhanced translation of BACE1, a mechanism demonstrated in neuronal and retinal models exposed to oxidative challenge.^88^ Preclinical studies further show that even sub-toxic concentrations of mitochondrial inhibitors markedly elevate retinal BACE1 expression and enzymatic activity in the absence of cell death, underscoring the extreme sensitivity of BACE1 to mitochondrial redox imbalance.^36^ In parallel, mitochondrial dysfunction promotes cytochrome c release and caspase activation, initiating apoptotic pathways that contribute to synaptic degeneration and retinal ganglion cell loss, processes that are exacerbated by amyloid-β species generated through BACE1 activity.^89^ Importantly, diabetes-induced mitochondrial DNA (mtDNA) damage and reduced mitochondrial biogenesis, including downregulation of PGC-1α and associated transcriptional regulators, further impair the oxidative capacity of retinal cells.^21^ Together, these abnormalities create a pathological feedback loop in which mitochondrial dysfunction accelerates BACE1 activation, and BACE1-derived amyloidogenic stress further damages mitochondria, thereby amplifying neurodegenerative and vascular injury in DR. In this study, we have confirmed the role of BACE1 and its relation with retinal function in DR patients before and after CoQ10 supplement for 12 weeks.

 A seminal study by Xiong et al. reveals that oxidative stress, whether initiated by mitochondrial dysfunction or exposure to exogenous pro-oxidative agents, directly amplifies BACE1 protein levels, enzymatic activity, and amyloidogenic processing within the retina.^36^ This mechanistic link was demonstrated through the use of mitochondrial electron transport chain inhibitors targeting complexes I, II, and IV, each of which markedly elevated retinal BACE1 expression and increased production of its neurotoxic cleavage product, Aβ40, even at relatively low micromolar concentrations. Notably, oxidative stressors intrinsic to neurodegenerative pathology, such as ferric iron (Fe³⁺) and Aβ42 fibrils (Aβ42f), also produced robust increases in BACE1 activity following intravitreal administration, further reinforcing the sensitivity of this pathway to oxidative imbalance. Remarkably, Aβ42f induced significant BACE1 upregulation at intraocular concentrations as low as ~1 nM, underscoring the enzyme’s extreme vulnerability to oxidative insults and its capacity to respond to very subtle perturbations in redox homeostasis.^36^ Importantly, the elevation of BACE1 occurred even at subtoxic doses of mitochondrial inhibitors ( ≤ 10 μM) that failed to provoke indicators of structural injury such as GFAP-mediated glial reactivity or overt neuronal damage. This finding highlights that oxidative stress itself—rather than secondary cellular breakdown, mitochondrial energy depletion, or neuroinflammation—is the primary initiator of BACE1 dysregulation in retinal tissue.^90^

 Once upregulated, BACE1 catalyzes the amyloidogenic cleavage of amyloid precursor protein (APP), leading to the generation and accumulation of amyloid-β peptides (Aβ40/42) within retinal neurons, endothelial cells, and pericytes. These Aβ species contribute to mitochondrial depolarization, disturb intracellular calcium homeostasis, and promote synaptic dysfunction, ultimately impairing retinal ganglion cell viability and disrupting visual signal processing.^91^ Beyond their neurodegenerative effects, Aβ aggregates propagate vascular pathology through oxidative-stress–driven upregulation of VEGF, thereby enhancing vascular permeability, promoting blood–retinal barrier breakdown, and exacerbating the microvascular abnormalities characteristic of DR.^92^ Collectively, these interactions establish a self-reinforcing pathological feed-forward loop in which oxidative stress induces BACE1 activation, BACE1 augments Aβ production, and Aβ further amplifies oxidative stress, inflammation, and vascular injury. This cyclic mechanism underscores the interconnected nature of oxidative, amyloidogenic, and inflammatory pathways in DR, and highlights BACE1 as a central molecular node linking mitochondrial dysfunction to neurovascular degeneration.

 The selective vulnerability of synaptic BACE1 isoforms to oxidative stress underscores a fundamental mechanism underlying early amyloidogenic pathology in metabolically active retinal neurons.^93^ Although mitochondrial inhibitors broadly generate oxidative stress, their ability to enhance BACE1 expression closely mirrors the effects of direct pro-oxidants such as Fe³⁺ and Aβ42 fibrils (Aβ42f), which, at higher concentrations, induce laminar disruption, synaptic loss, and glial activation.^94^ This convergence of effects across mechanistically distinct stimuli highlights oxidative stress as a unifying upstream driver of BACE1 induction, regardless of whether the insult originates from mitochondrial dysfunction, transition metals, or amyloidogenic species. The tight interdependence between oxidative stress and BACE1 activity becomes even more apparent when considering Aβ42’s dual role: as a product of BACE1-mediated APP cleavage, Aβ42 aggregates increase intracellular oxidative stress, and this amplified oxidative burden feeds back to further enhance BACE1 expression. Such reciprocity establishes a self-sustaining pathological loop that is especially detrimental in neurodegenerative conditions like diabetic retinopathy, where chronic metabolic imbalance perpetuates oxidative injury.

 Consistent with this model, retinal BACE1 elevations observed in diabetic animal models closely parallel responses to hydrogen peroxide and Aβ42 exposure in vitro, reinforcing the conclusion that oxidative stress functions as a central regulator of BACE1 activity. Nevertheless, the intricate interplay among mitochondrial dysfunction, oxidative damage, and BACE1 activation complicates efforts to determine the initial triggering event in DR. For example, mitochondrial deficits may precede and initiate BACE1 upregulation, yet the subsequent accumulation of Aβ42 significantly exacerbates oxidative stress, thereby intensifying neuronal injury and accelerating vision loss.^36^ This cyclical pattern blurs traditional distinctions between cause and consequence in DR progression, emphasizing the dynamic and interconnected nature of the underlying molecular processes. Taken together, oxidative stress emerges as a critical nexus linking mitochondrial impairment to BACE1 dysregulation in the diabetic retina. The extreme sensitivity of synaptic BACE1 isoforms to even subtle redox imbalances, combined with Aβ42’s capacity to amplify oxidative damage, establishes a pathogenic feedback loop with profound implications for retinal neurodegeneration. Therapeutic strategies that target the oxidative stress–BACE1 axis hold considerable promise for interrupting this cycle and mitigating amyloidogenic cascades in DR. Therefore, in relation to the previously discussed antioxidant effects of CoQ10, one plausible mechanism contributing to the observed reduction in BACE1 activity among DR patients receiving CoQ10 supplementation may be the compound’s ability to restore redox balance and attenuate oxidative stress, thereby disrupting this harmful feedback loop. Exact and detailed mechanisms remains to be elucidated.

## Limitations

 This study has several limitations that should be acknowledged. First, the mechanistic scope of the trial was restricted to serum biomarkers. Although BACE1 activity was measured, downstream molecular pathways, including amyloid-β isoforms, VEGF signaling, retinal neurodegeneration markers, and gene-expression profiles, were not assessed due to the substantial financial and laboratory resources required for such analyses. As a result, the study provides correlative rather than causal mechanistic insight.

 Second, ophthalmological imaging outcomes such as OCT/OCTA measurements and DR grade progression were not included. The cost of retinal imaging alone exceeded the entire budget allocated for this student-level project. Therefore, the study relies primarily on biochemical markers, supplemented by a single functional visual outcome (contrast sensitivity), which limits the ability to link biochemical improvements to structural retinal changes.

 Third, the intervention lasted 12 weeks, which may not be sufficient to evaluate the long-term sustainability, safety, or clinical durability of CoQ10 supplementation. Resource limitations, ethical considerations regarding participant burden, and the advanced age of the study population made longer follow-up infeasible.

 Fourth, although the sample size was determined a priori using G*Power and is comparable to or larger than many clinical studies in DR, it remains insufficient for detailed subgroup analyses. Recruiting DR patients who meet strict ophthalmic, metabolic, and cognitive criteria is inherently challenging, especially in older populations with multiple comorbidities.

 Fifth, the study population consisted exclusively of Iranian adults, potentially limiting external generalizability. Differences in dietary patterns, metabolic phenotypes, and genetic variations influencing CoQ10 absorption and utilization may affect the applicability of the findings to other populations.

 Finally, ethical and logistical considerations limited repeated sampling and prevented the inclusion of more invasive ocular assessments. These factors collectively restricted the scope of the mechanistic and clinical evaluations.

## Future Directions

 Future research should build upon these exploratory findings by incorporating comprehensive mechanistic analyses. Studies should evaluate downstream molecular pathways such as amyloid-β accumulation, VEGF modulation, neuroinflammatory markers, mitochondrial function indices, oxidative DNA damage markers (e.g., 8-OHdG), and BACE1 gene-expression profiles to better elucidate the biological role of BACE1 in diabetic retinopathy.

 Future clinical trials should also integrate structural and functional ophthalmic outcomes, including OCT/OCTA imaging, fluorescein angiography, DR grade progression, visual acuity, and contrast sensitivity, to determine whether biochemical improvements translate into meaningful retinal and visual benefits. Longer intervention periods ( > 6 months) and extended post-intervention follow-up are needed to assess the sustainability and safety of CoQ10 supplementation.

 Larger, multi-center studies across diverse ethnic and metabolic backgrounds are essential to improve external validity and examine how genetic, dietary, and pharmacokinetic differences influence CoQ10 responsiveness.

 Additionally, dose–response investigations, mechanistic animal studies, and cellular experiments targeting BACE1 pathways may provide deeper insight into the molecular mechanisms linking CoQ10 supplementation, oxidative stress, inflammation, and neurodegeneration. Collectively, these future studies will be critical for determining the therapeutic potential of CoQ10 and BACE1 modulation in the prevention and management of diabetic retinopathy.

## Conclusion

 The results of the current study illustrated that daily supplementation with 200 mg of CoQ10 for 12 weeks in patients with DR could lead to a significant improvement in values of CoQ10, TAC, GSH, and CAT, as well as a significant decrease in TOS, MDA, TNF-α, IL-6, CRP, and BACE1 activity. Additionally, there was a close correlation between BACE1 activity and the levels of CoQ10, oxidative stress, and inflammatory parameters. Apart from all these improvements, CoQ10 could enhance contrast sensitivity among the DR patients. Thus, it can be concluded that possibly, an enhancement in the antioxidant defense system and a reduction in inflammation might result in less BACE1 activity and a reduction in BACE1 activity led to an enhancement in visual function. However, further and more fundamental research is required to investigate the involved mechanisms in detail.

## Competing Interests

 The authors declare that they have no known competing financial interests or personal relationships that could have appeared to influence the work reported in this paper.

## Ethical Approval

 The study protocol was approved by the ethics committee of the Hamadan University of Medical Sciences (UMSHA, code: IR.UMSHA.REC.1400.969), and informed consent forms were signed by all participants before the intervention.
